# A Novel *POR* G88S Mutation Causes Severe PORD and Establishes a Critical Pharmacogenomic Risk Profile

**DOI:** 10.1210/clinem/dgaf630

**Published:** 2025-11-19

**Authors:** Maria Natalia Rojas Velazquez, Jimena Lopez Dacal, Flemming Steen Jørgensen, Nora Sanguineti, Katyayani Sharma, Roxana Marino, Natalia Pérez Garrido, Elisa Vaiani, Pablo Ramírez, Paula Scaglia, Agustín Izquierdo, Gabriela Sansó, María Gabriela Ropelato, Ignacio Bergadá, Rodolfo A Rey, Romina P Grinspon, Amit V Pandey

**Affiliations:** Department of Pediatrics, University Hospital Bern, Bern CH-3010, Switzerland; Department of Biomedical Research, University of Bern, Bern CH-3010, Switzerland; Graduate School for Cellular and Biomedical Sciences, University of Bern, Bern CH-3012, Switzerland; Centro de Investigaciones Endocrinológicas “Dr César Bergadá” (CEDIE), CONICET-FEI-División de Endocrinología, Hospital de Niños Ricardo Gutiérrez, C1425EFD Buenos Aires, Argentina; Department of Drug Design and Pharmacology, University of Copenhagen, Copenhagen DK-2100, Denmark; Centro de Investigaciones Endocrinológicas “Dr César Bergadá” (CEDIE), CONICET-FEI-División de Endocrinología, Hospital de Niños Ricardo Gutiérrez, C1425EFD Buenos Aires, Argentina; Department of Pediatrics, University Hospital Bern, Bern CH-3010, Switzerland; Department of Biomedical Research, University of Bern, Bern CH-3010, Switzerland; Graduate School for Cellular and Biomedical Sciences, University of Bern, Bern CH-3012, Switzerland; Servicio de Endocrinología, Hospital de Pediatría Juan P. Garrahan, C1245AAM Buenos Aires, Argentina; Servicio de Endocrinología, Hospital de Pediatría Juan P. Garrahan, C1245AAM Buenos Aires, Argentina; Servicio de Endocrinología, Hospital de Pediatría Juan P. Garrahan, C1245AAM Buenos Aires, Argentina; Servicio de Endocrinología, Hospital de Pediatría Juan P. Garrahan, C1245AAM Buenos Aires, Argentina; Centro de Investigaciones Endocrinológicas “Dr César Bergadá” (CEDIE), CONICET-FEI-División de Endocrinología, Hospital de Niños Ricardo Gutiérrez, C1425EFD Buenos Aires, Argentina; Unidad de Medicina Traslacional, Hospital de Niños Ricardo Gutiérrez, C1425EFD Buenos Aires, Argentina; Centro de Investigaciones Endocrinológicas “Dr César Bergadá” (CEDIE), CONICET-FEI-División de Endocrinología, Hospital de Niños Ricardo Gutiérrez, C1425EFD Buenos Aires, Argentina; Unidad de Medicina Traslacional, Hospital de Niños Ricardo Gutiérrez, C1425EFD Buenos Aires, Argentina; Centro de Investigaciones Endocrinológicas “Dr César Bergadá” (CEDIE), CONICET-FEI-División de Endocrinología, Hospital de Niños Ricardo Gutiérrez, C1425EFD Buenos Aires, Argentina; Unidad de Medicina Traslacional, Hospital de Niños Ricardo Gutiérrez, C1425EFD Buenos Aires, Argentina; Centro de Investigaciones Endocrinológicas “Dr César Bergadá” (CEDIE), CONICET-FEI-División de Endocrinología, Hospital de Niños Ricardo Gutiérrez, C1425EFD Buenos Aires, Argentina; Unidad de Medicina Traslacional, Hospital de Niños Ricardo Gutiérrez, C1425EFD Buenos Aires, Argentina; Centro de Investigaciones Endocrinológicas “Dr César Bergadá” (CEDIE), CONICET-FEI-División de Endocrinología, Hospital de Niños Ricardo Gutiérrez, C1425EFD Buenos Aires, Argentina; Centro de Investigaciones Endocrinológicas “Dr César Bergadá” (CEDIE), CONICET-FEI-División de Endocrinología, Hospital de Niños Ricardo Gutiérrez, C1425EFD Buenos Aires, Argentina; Unidad de Medicina Traslacional, Hospital de Niños Ricardo Gutiérrez, C1425EFD Buenos Aires, Argentina; Centro de Investigaciones Endocrinológicas “Dr César Bergadá” (CEDIE), CONICET-FEI-División de Endocrinología, Hospital de Niños Ricardo Gutiérrez, C1425EFD Buenos Aires, Argentina; Department of Pediatrics, University Hospital Bern, Bern CH-3010, Switzerland; Department of Biomedical Research, University of Bern, Bern CH-3010, Switzerland

**Keywords:** adrenal insufficiency, ambiguous genitalia, androgen synthesis defect, PORD, congenital adrenal hyperplasia, CY17A1

## Abstract

**Context:**

P450 oxidoreductase (POR) deficiency is a rare congenital adrenal hyperplasia with variable severity. The mechanisms of severe mutations and their full metabolic consequences, including drug metabolism, are not fully characterized.

**Objective:**

To define the clinical, biochemical, and molecular consequences of a novel homozygous *POR* missense mutation, p.Gly88Ser (G88S), identified in 4 unrelated Argentine families.

**Design:**

A translational study combining clinical case series analysis with comprehensive in vitro molecular and functional characterization of the novel protein variant.

**Setting:**

Tertiary pediatric endocrine centers in Argentina and Switzerland.

**Patients:**

We report 5 individuals (4 46,XY; 1 46,XX) from 4 unrelated families presenting with disorders of sex development and adrenal dysfunction.

**Main Outcome Measures:**

Clinical phenotypes, hormonal profiles, and *POR* gene sequencing. In vitro analysis of recombinant POR measured flavin content, reductase activity, and support of steroidogenic and drug-metabolizing P450s.

**Results:**

All patients were homozygous for the c.262G>A (p.G88S) mutation. This FMN binding domain variant caused protein instability with severe loss of FMN (<30%) and FAD (<15%) cofactors. Steroidogenic activities were virtually abolished (CYP21A2: 1.3%; CYP17A1 17,20-lyase: 5.5% of wild-type), explaining the clinical phenotype. Activities of major drug-metabolizing enzymes were also severely impaired (3%-9% of wild-type), establishing a “poor metabolizer” phenotype.

**Conclusion:**

The *POR* G88S mutation causes one of the most severe forms of PORD described, driven by dynamic protein instability and cofactor loss. It is a critical pharmacogenomic marker, and its recurrence in Argentina suggests a potential screening target.

Cytochrome P450 oxidoreductase (POR) deficiency (PORD), with OMIM designations 613571 and 201750, is a type of congenital adrenal hyperplasia that was initially discovered in patients exhibiting abnormal steroidogenesis (1, 2). In 2004, we and others described mutations in the *POR* gene that caused alterations in steroid metabolism (2-6). Further investigations have revealed that many *POR* mutations result in disorders of sexual development, which are sometimes accompanied by bone malformation (7-11). POR is crucial for transferring redox equivalents from the reduced form of nicotinamide adenine dinucleotide phosphate (NADPH) to type 2 cytochrome P450 proteins located in the endoplasmic reticulum, which allows them to perform their catalytic functions (Fig. 1A) (12, 13). Furthermore, POR has the ability to reduce heme oxygenase, cytochrome b_5_, and a variety of small molecules and dyes (Fig. 1B) (14-22). POR's involvement in many metabolic processes makes it essential for life, as shown by the fact that POR knockout mice die during embryonic development (23-29). The wide range of disorders linked to deficiency of POR highlights the critical role of POR in various physiological processes. The effect of POR mutations on steroid metabolism can result in a range of endocrine and reproductive issues (30-32). Additionally, POR's involvement in other metabolic pathways may contribute to the diverse clinical manifestations seen in patients with PORD. To fully explain the complex role of POR in health and disease, and to develop targeted therapies for PORD, further research is required (33, 34).

**Figure 1.**
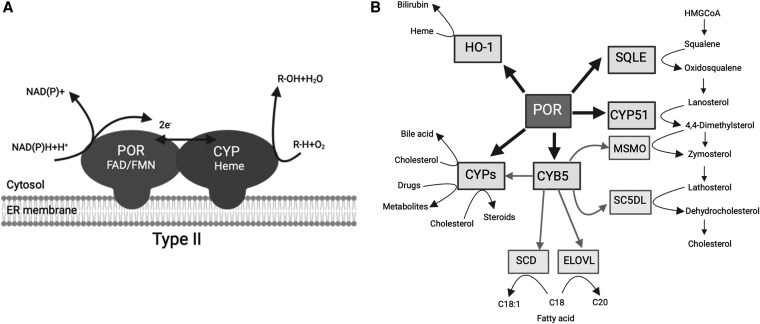
Role of POR in metabolism. A, Schematic representation of electron transfer from P450 oxidoreductase (POR) to a Type II cytochrome P450 (CYP) enzyme in the endoplasmic reticulum (ER) membrane. The FMN domain of POR interacts with a cytochrome P450 (CYP) enzyme, which is also embedded in the ER membrane. B, The central role of P450 oxidoreductase (POR) in human metabolic processes. POR is crucial for the activity of several CYPs involved in the synthesis of all steroid hormones, including those of the adrenal cortex (eg, cortisol, aldosterone, androgens) and the gonads (eg, estrogens, testosterone). POR supports CYP51 (lanosterol synthase), for the conversion of lanosterol to cholesterol in the cholesterol biosynthesis pathway. POR may also interact with MSMO (methylsterol monooxygenase) in further steps of this pathway. POR is also the obligate electron donor for many hepatic and extrahepatic CYPs, which are responsible for the phase I metabolism of a vast number of drugs, toxins, and other xenobiotic compounds. POR also interacts with heme oxygenase-1 (HO-1) in the breakdown of heme, for the formation of bilirubin and in conjunction with cytochrome b_5_ (CYB5), provides electrons to enzymes like Stearoyl-CoA Desaturase (SCD) and enzymes involved in the elongation of Very Long-Chain Fatty Acids (ELOVL), which are critical for the synthesis of unsaturated and long-chain fatty acids. Dysfunction of POR, in POR deficiency, can therefore lead to a wide spectrum of clinical manifestations affecting these diverse metabolic pathways. Created in BioRender. Pandey, A. (2025) https://BioRender.com/xyv6go9.

The POR protein is critical to the cytochrome P450 system, as it interacts with 50 different cytochrome P450 proteins (1, 35, 36). Due to this extensive network of interactions, it is difficult to accurately predict the consequences of specific POR mutations (37-42). However, extensive research has shown some trends. Mutations that result in the loss of flavin cofactors (flavin adenine dinucleotide [FAD] and flavin mononucleotide [FMN]) within the POR protein have a stronger effect on enzymatic activity (1, 43-46). This loss causes a severe form of PORD that is often lethal. While protein instability is a known pathomechanism for some severe *POR* mutations, the specific molecular dynamics leading to such instability and catastrophic cofactor loss are not fully understood for all variants (31, 47, 48). The hinge region of the POR protein is essential for the movement of the FMN and FAD domains (49-54). Mutations in this region can cause a form of PORD with a wide range of symptoms (47). The severity and specific manifestations of the disease can depend on which P450 enzymes are involved and the extent to which they are affected. The NADPH binding site is another crucial region of the POR protein. Mutations near this site can disrupt the activity of multiple cytochrome P450 enzymes (30, 55). The specific effects on individual enzymes can differ, leading to a complex array of symptoms and varying disease severity. Overall, *POR* mutations can have a significant effect on the human cytochrome P450 system, resulting in a range of disorders with varying degrees of severity. Due to the complexity of the POR protein's interactions with numerous cytochrome P450 proteins, the specific effects of individual mutations are difficult to predict. However, ongoing research continues to clarify the underlying mechanisms and potential therapeutic targets for POR-related disorders (56).

In the current study, a recently discovered c.262G>A (p.Gly88Ser [G88S]) mutation in *POR* was investigated. This mutation was identified in several unrelated patients from Argentina who exhibited impaired gonadal and adrenal steroid production. The multiple enzyme activities affected by the G88S mutation in *POR* were characterized through biochemical analysis of patient samples and the generation of recombinant POR protein incorporating the G88S mutation. We focused on the effects of the G88S mutation on 2 steroid-metabolizing P450 enzymes, CYP17A1 (17-hydroxylase and 17,20 lyase activities) and CYP21A2 (21-hydroxylase activity) based on biochemical laboratory results and hormonal measurements from patients. We confirmed that the G88S mutation severely impaired CYP17A1 and CYP21A2 activities, which explains the under-virilization in 46,XY individuals and the pubertal delay in one 46,XX patient associated with adrenal insufficiency. Furthermore, given that PORD is known to affect drug metabolism, we investigated the quantitative impact of this severe mutation on a broad panel of drug-metabolizing enzymes, an under-investigated area with critical implications for the lifelong clinical management of affected individuals. The direct metabolic activities of POR on small molecules, and a soluble probe substrate cytochrome c were also tested to identify different mechanisms that may affect POR due to the G88S mutation. The experiments revealed 2 distinct mechanisms for the pathogenicity of the *POR* G88S mutation: The inhibition of CYP17A1 and CYP21A2 activities and an overall reduction in POR activities due to a loss of flavin content, suggesting protein instability. These findings provide valuable insights into the functional consequences of the G88S mutation in *POR* and its contribution to the insufficient gonadal and adrenal steroid hormone production in affected individuals. By elucidating the molecular mechanisms underlying POR-related disorders, this research may facilitate the development of novel diagnostic and therapeutic approaches for patients with *POR* mutations.

## Methods

### Study Approval and Patient Consent

The study was conducted in accordance with the Declaration of Helsinki and was approved by the Ethics Review Committee of the Hospital de Niños Ricardo Gutiérrez, Buenos Aires, Argentina. Written informed consent was obtained from the parents or legal guardians of all participants.

### Clinical Assessment

Clinical assessments included length and weight measurements expressed as SD scores based on the Argentine population reference (57), external genitalia scoring (EGS) (58), pubertal staging according to Marshall and Tanner system (59), and penile size measurement compared to population-specific data of the Argentine population (60).

### Hormone Assays

Serum levels of follicle-stimulating hormone (FSH), luteinizing hormone (LH), testosterone, progesterone, and cortisol were measured by electrochemiluminescence assay (ECLIA) on a COBAS e-411 analyzer (Roche Diagnostics). Dehydroepiandrosterone sulfate (DHEA-S) and adrenocorticotropin (ACTH) were measured by chemiluminescence assay on an IMMULITE 2000XPi analyzer (Siemens). Androstenedione (A) and 17-hydroxyprogesterone (17OHP) were measured by radioimmunoassay (RIA) (Diasource) (61, 62). Anti-Müllerian hormone (AMH) was measured by enzyme-linked immunosorbent assay (ELISA) (Immunotech-Beckman) Marseilles, France) (63, 64). For detailed assay performance, including sensitivities and coefficients of variation, see Supplementary Methods at https://doi.org/10.48620/91916 (65). ACTH (250 µg/m², max 250 µg) and human chorionic gonadotropin (hCG) stimulation test consisted of 6 injections of 1500 IU or 4 injections of 2500 IU every other day (66). Testosterone, androstenedione, 17OHP, and progesterone were measured before stimulation and one day after the final injection (67).

### Next-Generation Sequencing and Filtering

Genomic DNA was extracted from peripheral venous blood cells and DNA library preparation and exon capture from the proband were performed using the TruSight One® sequencing panel (Illumina), The quality of genomic DNA fragmentation was evaluated using a capillary system Fragment Analyzer^TM^ (Advanced Analytical). Next-generation sequencing (NGS) was performed using a NextSeq 500® system (Illumina) at the Translational Medicine Unit of the Buenos Aires Children's Hospital (Unidad de Medicina Traslacional, Hospital de Niños Ricardo Gutiérrez, Buenos Aires).

We used the strategy recommended by the Broad Institute in the Genome Analysis Toolkit (GATK Best Practices^TM^) for preprocessing, variant calling, and refinement. Raw sequence data were mapped to the 1000-Genomes phase II reference genome (GRCh37 version hs37d5) using the BWA-MEM algorithm of Burrows-Wheeler Aligner software (68) and visualized with the Integrative Genomics Viewer (IGV v.2.12.3). Duplicates were removed using Picard (Broad Institute). The variant call format file (VCF) was annotated following ANNOVAR protocol (69) with the following databases: ClinVar (https://www.ncbi.nlm.nih.gov/clinvar/) (70), gnomAD (https://gnomad.broadinstitute.org/) (68), dbSNP (https://www.ncbi.nlm.nih.gov/snp/) (71), GWAS Catalog (https://www.ebi.ac.uk/gwas/) (72), InterVar (https://wintervar.wglab.org/) (69), among others. Copy number variants were evaluated using DECoN algorithm prediction (73).

Variant filtering and prioritization were performed on B_platform (https://www.bitgenia.com/b-platform/). The candidate gene list, according to the patient’s 46,XY difference/disorder of sex development (DSD) phenotype, included the following genes: *AKR1C2, AKR1C4, ARX, ATRX, CDKN1C, CYB5A, CYP11B1, CYP17A1, CYP19A1, CYP21A2, DHCR7, ESR1, FGFR2, HSD17B3, HSD17B4, HSD3B2, LHCGR, NR3C1, POR, SRD5A2, STAR*. Variants were classified according to their potential pathogenicity using the American College of Medical Genetics and Genomics (ACMG) guidelines for variant interpretation (69) following the ClinGen Sequence Variant Interpretation working group recommendations (https://www.clinicalgenome.org/working-groups/sequence-variant-interpretation/).

### Sanger Sequencing

Genomic DNA was isolated from peripheral blood leukocytes by conventional methods and coding region (exons 1-15) and splice sites in flanking intronic regions of the POR gene were amplified by polymerase chain reaction (PCR) and sequenced using BigDye Terminator version 3.1 cycle sequencing kit (Applied Biosystems, Buenos Aires, Argentina) on an ABI PRISM® 3130 Genetic Analyzer capillary DNA sequencer (Applied Biosystems, Buenos Aires, Argentina). The primers used for sequencing were the same as those used for PCR and are listed in Supplementary Table S1 available at https://doi.org/10.48620/91916 (65). Previously reported intronic mutations were also analyzed (Human Gene Mutation Database [HGMD], www.hgmd.cf.ac.uk/). The nucleotide sequences obtained were compared with the NCBI entries of POR gene (NG_008930.1) using SeqScape software v0.2.6 (Applied Biosystems).

### Expression of POR Proteins in Bacteria and Membrane Purification

The human POR-WT and G88S mutant proteins (NCBI# NP_000932, Uniprot# P16435) were expressed in bacteria utilizing a heterologous gene expression protocol. Recombinant POR variants (N-23 form) were expressed, and bacterial membranes were purified based on previous publications (8, 31, 74, 75).

### Western Blot Analysis to Determine POR Content in Membranes

Western blot analysis was conducted to determine POR content in membranes as described previously (30, 76). In short, 1 μg of bacterial membrane proteins were separated on an SDS-PAGE gel, blotted onto polyvinyl difluoride (PVDF) membranes, and probed with a rabbit polyclonal antibody against POR-WT from Genscript (Genscript, NJ, USA) followed by a secondary goat anti-rabbit antibody labeled with an infrared dye (IRDye 680RD, LI-COR Bioscience Inc., NE, USA). Purified wild-type (WT) POR was used as a standard for normalizing the POR content of each membrane preparation. The normalized amount of POR content was used for POR-WT and POR-G88S protein in all the experiments described here.

### Small Molecule Reduction Assay by POR-WT and POR-G88S

#### Cytochrome C assay

To evaluate cytochrome c reduction activity, triplicate reactions were conducted in a 96-well plate using a microplate reader. The reaction mixture comprised 50nM POR in 50mM Tris-HCl buffer (pH 7.8), 150mM NaCl and different concentrations of cytochrome c (2.5μM to 60μM). The reaction was initiated by introducing 100µM NADPH, and the absorbance at 550 nm was monitored for 10 minutes using a Spectramax M2e plate reader (Molecular Devices, Sunnyvale, CA, USA). Vmax and Km values were obtained by fitting the data to the Michaelis-Menten equation (31, 33).

#### NADPH-dependent MTT assay

For the NADPH-dependent MTT (3-(4,5-dimethylthiazol-2-yl)-2,5-diphenyltetrazolium bromide) assay, the reaction mixture contained 50nM POR in 50mM Tris-HCl buffer (pH 7.8) with 150mM NaCl and different concentrations of MTT (3.9µM to 500µM). The reaction was initiated by adding 100µM NADPH, and the rate of increase in absorbance at 610 nm was measured for 30 minutes using the extinction coefficient (ε610 = 11 mM^−1^ cm^−1^); And Vmax and Km values were obtained by fitting the data to the Michaelis-Menten equation (31, 33).

#### NADPH-dependent resazurin reduction assay

For NADPH-dependent resazurin (RS) reduction assay, the reaction mixture consisted of 100nM POR in 50mM Tris-HCl buffer (pH 7.8) with 150mM NaCl with variable concentrations of resazurin (2.5µM to 20µM) and the rate of increase in emission (570 nm excitation, 585 nm emission for RS) was measured using an extinction coefficient (ελ = 60 mM^−1^ cm^−1^) on a Spectramax M2e plate reader (Molecular Devices, Sunnyvale, CA, USA). Vmax and Km values were obtained by fitting the data to the Michaelis-Menten equation (31, 33).

#### Flavin content analysis of WT and mutant POR

To determine the flavin content of the protein samples, the samples were first treated with 2M urea for 1 hour to release the flavin molecules from the protein structure. The solution was then centrifuged at 13 000 rpm for 10 minutes to remove any precipitated proteins. The fluorescence intensity of the released flavin molecules, flavin mononucleotide (FMN) and flavin adenine dinucleotide (FAD), was then measured separately at pH 7.7 and pH 2.6, respectively, with excitation at 450 nm and emission at 535 nm (77).

#### Assay of CYP17A1 activities

The 17α-hydroxylase activity of CYP17A1 was assayed using [¹⁴C]-progesterone as the substrate (2, 8, 78, 79) using pure wild-type/mutant POR, purified CYP17A1 (obtained from CYPEX, Dundee, Scotland, UK), and cytochrome b₅ in a ratio of 4:1:1. Steroids were extracted, separated by thin-layer chromatography (TLC) (silica gel 60 F-254 TLC plates, Merck) using ethyl acetate:chloroform (3:1) as the solvent system, and quantified by phosphorimaging (8, 31, 74, 75). The CYP17A1 17,20 lyase activity was measured by the release of tritiated water from [21-³H]-17-OH-pregnenolone substrate as described (31, 33, 37, 80-83).

#### Assay of CYP21A2 activity

The 21-hydroxylase activity of CYP21A2 was assayed using [¹⁴C]-progesterone as the substrate (84-87). Reactions were initiated by adding 1mM NADPH and were stopped with ethyl acetate:isooctane (3:1). Steroids were extracted, separated by TLC (silica gel 60 F-254 TLC plates, Merck) using ethyl acetate:chloroform (3:1) as the solvent system, and quantified by phosphorimaging.

#### Assay of CYP2C9, CYP2C19, CYP3A4, and CYP3A5 activities

The activities of CYP2C9, CYP2C19, CYP3A4, and CYP3A5 supported by wild-type (WT) or mutant POR were assessed using an in vitro reconstituted system (30, 37, 47, 55, 76, 88-90). For CYP2C9, fluorogenic compound BOMCC was utilized as a substrate. For CYP2C19, 20µM EOMCC was used as a substrate. The CYP3A4/CYP3A5, assay used WT or G88S POR, purified CYP3A4, and cytochrome b_5_ at a ratio of 5:1:1 and 20 μM BOMCC as substrate. To initiate the reaction, 1mM NADPH was added, and the progression of the reaction was monitored by a fluorescence spectrophotometer with sample excitation at 415 nm and emission at 460 nm. All assays utilized a Spectramax M2e plate reader to measure fluorescence, and all reactions were initiated by the addition of NADPH.

### Molecular Modeling

Structures of the closed conformation of the human POR (PDB 3QE2) and open conformation of the rat POR (PDB 3ES9) were extracted from the Protein Data Bank (91, 92). The glycine to serine mutations were done in Maestro using the Mutate Residues procedure (https://www.schrodinger.com/platform/products/maestro/). All structures were prepared for the molecular dynamics (MD) simulations using the Protein Preparation Procedure in Maestro. MD simulations were performed by the Desmond program accessed from Maestro and applying the default setup procedure. The POR closed conformations were simulated for 100 ns, the FMN domains were simulated for 500 ns, for both systems 100 frames were collected and analyzed. Figures of structures were prepared in PyMol.

### Statistical analysis

The data were presented as mean and standard error of the mean (SEM). Student's *t* test was used to analyze the differences within experimental subsets; *P* values less than .05 were considered to be statistically significant.

## Results

### Case Reports

Patient 1 was referred at 2 months of life for ambiguous genitalia. Parents were reportedly non-consanguineous of mixed European-Argentine origin, and there was no remarkable family history. The patient was born at 39 weeks with appropriate weight for gestational age and presented imperforate anus and colo-vesical fistula that required early surgical repair. The patient had been assigned male. External genital score (EGS) was 9/12, with phallus of 0.5 cm long and 1 cm wide, distal urethral meatus, and fused labioscrotal folds with bilateral palpable gonads. No skeletal features of Antley-Bixler syndrome were observed. Blood pressure was normal. Karyotype was 46,XY. No Müllerian ducts were present on ultrasonography. At 2 months of age, basal testosterone was low (18 ng/dL, ref 180-270), with normal LH (0.25 U/L, ref 0.20-4.20) and FSH (3.8 U/L, ref 0.3-4.7). AMH was within normal male reference (385 pmol/L, ref 242-1583). At 3 months, Leydig cell testosterone secretion in response to hCG (4 × 2500 IU) was insufficient (Table 1). At 6 months, basal ACTH level was elevated with normal cortisol and high 17OHP and progesterone, whereas cortisol showed no response to ACTH stimulus. Altogether these observations were indicative of impaired CYP17A1 and CYP21A2 activities. With the presumptive diagnosis of 46,XY DSD due to a disorder of androgen synthesis, NGS was performed (see below). At the age of 13 years, the patient showed clear signs of primary hypogonadism: LH (18.9 IU/L) and FSH (10.1 IU/L) were high while testosterone was undetectable (<10 ng/dL). Additionally, he presented developmental delay and obesity. He did not require corticosteroid replacement but antistress guidelines were provided (Table 2). Our analysis confirmed that no pathogenic or likely pathogenic variants were found in the 241 Neurodevelopment-related genes included in the TruSight One® sequencing panel used for this case: *ACTB, ACTG1, ADNP, ADSL, AGA, AHDC1, ALDH5A1, ALDH7A1, AMER1, ANKRD11, AP1S2, ARG1, ARID1A, ARID1B, ARSA, ARX, ASNS, ASXL1, ATP1A3, ATP7A, ATRX, AUTS2, BCAP31, BRAF, BRAT1, BRD4, BRWD3, CACNA1A, CACNA1E, CAMK2B, CASK, CBL, CC2D2A, CDK13, CDKL5, CHD2, CHD7, CHD8, CLCN4, CLN2 (TPP1), CLN3, CLN5, CLN6, CLTC, CNTNAP2, COL4A1, CREBBP, CTNNB1, CUL3, DDC, DDX3X, DEAF1, DHCR7, DNM1L, DNMT3A, DOCK6, DPF2, DYNC1H1, DYRK1A, EEF1A2, EFTUD2, EHMT1, EP300, EZH2, FGD1, FOLR1, FOXG1, FOXP1, GABBR2, GABRB3, GABRG2, GALC, GAMT, GATAD2B, GATM, GLB1, GM2A, GNAO1, GNAS, GNS, GPC3, GRIA3, GRIN1, GRIN2A, GRIN2B, HDAC8, HEXA, HEXB, HGSNAT, HIVEP2, HNRNPK, HNRNPU, HRAS, HUWE1, IDS, IDUA, IGF1R, IL1RAPL1, IQSEC2, ITPR1, KANSL1, KAT6A, KAT6B, KCNA2, KCNB1, KCNH1, KCNQ2, KCNT1, KDM5C, KDM6A, KIF1A, KMT2A, KMT2B, KMT2D, KMT2E, KRAS, L1CAM, LZTR1, MAGEL2, MAN1B1, MAP2K1, MAP2K2, MBD5, MECP2, MED12, MED13L, MEF2C, MFSD8, MID1, MTOR, NAA10, NAA15, NAGLU, NALCN, NEXMIF, NF1, NFIA, NFIX, NGLY1, NHS, NIPBL, NONO, NPC1, NR2F1, NRAS, NRXN1, NSD1, NSUN2, OCRL, OPHN1, OTC, PACS1, PACS2, PAH, PCBD1, PCDH19, PDHA1, PGAP3, PHF21A, PHF6, PHIP, PLA2G6, PMM2, POLG, PPM1D, PPP1CB, PPP2R1A, PPP2R5D, PPP3CA, PPT1, PQBP1, PTEN, PTPN11, PTS, PURA, QDPR, RAD21, RAF1, RAI1, RBM10, RIT1, RPS6KA3, SATB2, SCN1A, SCN1B, SCN2A, SCN3A, SCN8A, SETBP1, SETD5, SGSH, SHOC2, SIN3A, SLC13A5, SLC16A2, SLC2A1, SLC6A1, SLC6A8, SLC9A6, SMARCA2, SMARCA4, SMARCB1, SMARCE1, SMC1A, SMC3, SON, SOS1, SOS2, SOX11, SPAST, SPATA5, SPTAN1, STAG1, STXBP1, SURF1, SYNGAP1, TAF1, TBCK, TBL1XR1, TCF20, TCF4, TELO2, TRAPPC9, TRRAP, TSC1, TSC2, TUBA1A, UBE3A, UNC80, USP9X, VPS13B, WDR45, WWOX, ZBTB18, ZBTB20, ZC4H2, ZDHHC9, ZEB2, ZIC2, ZMIZ1, ZMYND11*. Therefore, it is concluded that the homozygous *POR* G88S mutation is the only identifiable monogenic cause of this patient's primary phenotype. The additional features may be attributable to the known variable expressivity of severe PORD or potentially other complex genetic or environmental modifiers that are beyond the scope of this analysis.

**Table 1.** Hormonal values in index Patients 1 to 4 with POR deficiency. For patient 2 both post hCG and post ACTH tests are shown at 12 year of age

	Patient 1				Patient 2				Patient 3		Patient 4			
Age	3 months		6 months		12 yr		12 yr		10 yr		8 months		11 months	
**Hormonal test**	basal	Post hCG	basal	Post ACTH	basal	Post hCG	basal	Post ACTH	basal	Post ACTH	basal	Post hCG	basal	Post ACTH
**T ng/dL**	18	54	—	—	<10	156	<10	—	<20		40	95	<10	<10
**A4 ng/dL**	—	16	19	49	21	23	21	26	<10	<10	51	11	21	17
**17OHP ng/mL**	7.9	6.1	19.5	19.4	12.7	9.8	12.7	15	17.6	16.6	9.6	9.6	11.9	14.5
**Progesterone ng/mL**	21	25	26	> 66	20.5	10.7	20.5	26.3	34	> 66	—	—	> 66	> 66
**DHEA-S ng/mL**					591	304	591	588	<20		<150	<150	<150	<150
**ACTH pg/mL**	—	—	84	—	—	—	72	—	129		—	—	413	—
**Cortisol µg/dL**	—	—	11.1	11.1	—	—	9.3	8.5	5.7	4.7	—	—	8.3	8.5
**AMH pmol/L**	385				496	—	—	—	162		1328	—	—	—
**LH IU/L**	0.3	—	—	—	1.6	—	—	—	0.6		5.2	—	—	—
**FSH IU/L**	1.1	—	—	—	1.4	—	—	—	5.8		1.8	—	—	—

Abbreviations: 17OHP, 17-hydroxyprogesterone, A4, androstenedione, ACTH, adrenocorticotropic hormone, AMH, anti-Müllerian hormone, DHEA-S, dehydroepiandrosterone sulfate, FSH, follicle-stimulating hormone, LH, luteinizing hormone, T, testosterone.

**Table 2.** Patient clinical profiles

Patient	Age at presentation	Karyotype	Clinical features (DSD, puberty status, adrenal symptoms)	Associated malformations
Patient 1	2 months	46,XY	Ambiguous genitalia (EGS 9/12), imperforate anus, colo-vesical fistula. At 13 years: primary hypogonadism, developmental delay, obesity.	No skeletal features of ABS observed.
Patient 2	18 days	46,XY	Ambiguous genitalia (EGS 7/12), perineal hypospadias. At 12 years: no clinical signs of puberty. Requires sex steroid and corticosteroid replacement.	No skeletal features of ABS; normal bone X-ray imaging.
Patient 2's Sister	12.5 years	46,XX	Delayed puberty (Tanner B2 breast). Requires sex steroid hormone replacement.	Not reported.
Patient 3	9 years	46,XY	Prepubertal (Tanner B1, PH1), undervirilized genitalia (clitoris <1 cm). Requires hydrocortisone and sex steroid replacement.	Arachnodactyly, thoracic kyphosis, unilateral kidney agenesis, dysmorphic facial features (bulbous nose, flat nasal bridge) consistent with ABS.
Patient 4	5 days	46,XY	Ambiguous genitalia (EGS 4.5/12), perineoscrotal hypospadias. Adequately responded to testosterone administration.	Bulbous nose, left preauricular skin tags. No skeletal features of ABS.

A quick overview of the key clinical features of the patients with the homozygous G88S mutation in the *POR* gene.

Abbreviations: ABS, Antley-Bixler syndrome; difference/disorder of sex development; EGS, external genitalia scoring.

Patient 2, born at 39 weeks with appropriate weight and height for gestational age, was referred at 18 days of life for ambiguous genitalia. Parents reported an absence of consanguinity; the origin of both families was from Argentina. There was no significant family history. EGS was 7/12: the phallus was 1 cm long and 0.6 cm wide, and perineal hypospadias and fused labioscrotal folds with 3-mL palpable gonads were observed. Blood pressure was normal. Karyotype was 46,XY. No Müllerian ducts were present on ultrasonography. Neonatal screening revealed a slightly elevated 17OHP concentration, which was confirmed in serum after organic extraction (22.1 ng/mL, ref <3.8 ng/mL). However, the 17OHP concentration decreased to normal levels (2.5 ng/mL) by 45 days of life and remained within the normal range in later evaluations. At 18 days of life, the patient presented with high LH (24.8 IU/L, ref 0.4-7.6) and testosterone (433 ng/dL, ref 20-300), while FSH (3.1 IU/L, ref 0.3-4.1), and AMH (338 pmol/L, ref 242-1583) were within the male range. Partial androgen insensitivity was suspected, and male sex was assigned. The patient underwent hypospadias repair and received testosterone therapy to promote penile growth. At 12 years of age, the patient had no clinical signs of puberty, with a testicular volume of 3 mL bilaterally. LH, ACTH, progesterone, and 17OHP were high; cortisol, androstenedione, and testosterone were within the reference range for a prepubertal boy but relatively low for the elevated LH and ACTH levels (Table 1). The diagnosis was reassessed. Testosterone response to hCG (6 × 1500 IU) was borderline, and an absent response of cortisol to the ACTH test was observed. Altogether, these observations were suggestive of impaired CYP17A1 and CYP21A2 activities. Bone x-ray imaging was normal, with no indications of Antley-Bixler syndrome (Table 2). The patient required sex steroid and corticosteroid replacement therapy.

Patient 2's sister was evaluated at 12.5 years of age with breast Tanner stage 2. Blood pressure was normal. She presented with elevated basal ACTH (297 pg/mL, ref 10-46), 17OHP (16.7 ng/mL, ref 0.6-1.7), and progesterone (33.7 ng/mL, ref 0.4-1.1). Basal cortisol was within the reference values for age (11.7 µg/dL, ref 6-21). Estradiol was low (14 pg/mL, ref 20-77), with normal LH (4.7 IU/L, ref 0.1-5.3) and high FSH (8.7 IU/L, ref 1.1-7.3). Due to the lack of pubertal progression, sex steroid hormone replacement therapy was initiated.

Patient 3 was born from reportedly non-consanguineous parents of Argentine origin, at 39 weeks with appropriate weight and height for gestational age. The patient was raised as female and was referred to us at the age of 9 years. She had previously undergone urogenital sinus correction. The patient was prepubertal (Tanner staging B1, PH1), with a clitoris measuring less than 1 cm in length and a palpable left inguinal mass, consistent with a gonad. Additionally, she presented with arachnodactyly, thoracic kyphosis, unilateral kidney agenesis, and dysmorphic features, including a bulbous nose and a flat nasal bridge. Karyotype was 46,XY. No Müllerian ducts were detected on ultrasonography. Serum AMH (162 pmol/L) was elevated for female reference (5-55 pmol/L) but low for male reference (300-1800 pmol/L), indicating the existence of testicular tissue. Basal ACTH, progesterone, and 17OHP were high, whereas cortisol was low. Like in Patients 1 and 2, cortisol showed no response to the ACTH test (Table 1), suggesting impaired CYP17A1 and CYP21A2 function. Bilateral gonadectomy was performed, confirming the existence of testicular tissue without dysgenesis and supporting a steroid synthesis disorder. She required replacement therapy with hydrocortisone and sex steroids.

Patient 4 was delivered at 39 weeks with weight and height appropriate for gestational age and was referred at 5 days of life for ambiguous genitalia. The patient was the first child of reportedly non-consanguineous parents of mixed Argentine-European origin, and there was no significant family history. EGS was 4.5/12, with a phallus measuring 1 cm long and 0.3 cm wide, perineoscrotal hypospadias, partially fused labioscrotal folds, and bilateral inguinal gonads. Additionally, the patient had a bulbous nose and left preauricular skin tags. No skeletal features of Antley-Bixler syndrome were observed. Blood pressure was normal. Karyotype was 46,XY. No Müllerian ducts were observed on ultrasonography. At 19 days of life, testosterone was low (23 ng/dL, ref 20-300), with elevated LH (12.9 IU/L, ref 0.4-7.6) and normal AMH level (696 pmol/L, ref 242-1583) and FSH (3.8 IU/L, ref 0.3-4.1), suggesting a 46,XY DSD due to a disorder of androgen synthesis. Male sex was assigned. At 8 months of life, testosterone secretion in response to hCG (6 × 1500 IU) was insufficient (Table 1). Basal ACTH, progesterone, and 17OHP were high; basal cortisol was within the reference range but showed no changes after ACTH stimulation. An impaired CYP17A1 and CYP21A2 activity was suspected. The patient presented an adequate increase in penile size after testosterone administration. Surgical correction of hypospadias and bilateral orchidopexy were performed.

A critical observation from this case series is the variable expressivity of the Antley-Bixler syndrome skeletal phenotype. Despite sharing an identical homozygous G88S genotype, only Patient 3 presented with dysmorphic features consistent with Antley-Bixler syndrome, whereas the other 4 individuals did not have significant skeletal malformations (Table 2), which is in agreement with previous observations on PORD (93).

#### The POR G88S mutation is present in multiple unrelated Argentine families

**Figure 2.**
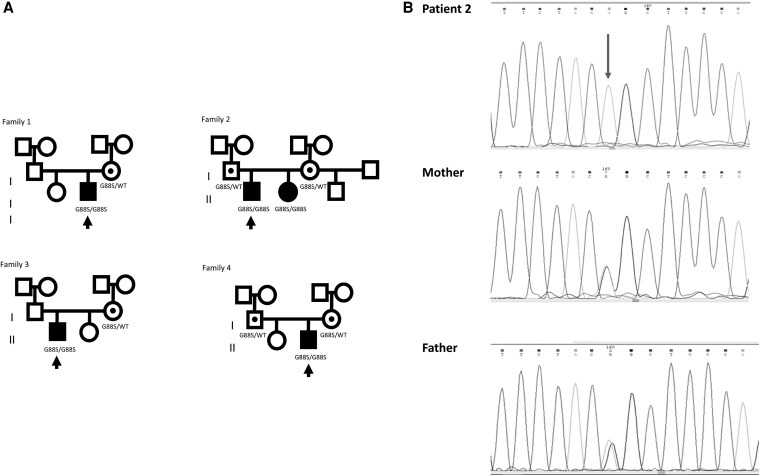
Pedigrees of 4 unrelated Argentine families with POR deficiency due to the G88S mutation. A, The family trees of 4 distinct families (Family 1, Family 2, Family 3, and Family 4) from Argentina, in which at least one individual was diagnosed with POR deficiency and found to be homozygous for the G88S mutation in the *POR* gene. In each pedigree, squares represent males, and circles represent females. Filled symbols indicate individuals affected by POR deficiency. The inheritance pattern observed in these families is consistent with autosomal recessive inheritance. B, Sanger sequencing analysis confirming the homozygous G88S mutation in Patient 2 and heterozygous carrier status in the parents. The top panel shows the chromatogram for Patient 2, revealing a homozygous guanine to adenine (G>A) transition at nucleotide position c.262 (indicated by the arrow). This mutation results in the substitution of glycine (Gly) to serine (Ser) at amino acid position 88 (p.Gly88Ser or G88S) in the POR protein. The middle and bottom panels show the chromatograms for the mother and father.

Targeted exome NGS in Patient 1 showed an average coverage of 72×, with ≥20× coverage in 92% of bases in the virtual gene panel. The initial analysis identified 27 952 variants in 4496 genes. Filtering for read depth ≥10× and GQ score ≥45, yielded 2040 variants in 1014 genes. Further analysis considering 21 candidate genes for 46,XY DSD available in the TruSight One sequencing panel and minor allele frequency (MAF) <1% in available population databases revealed only one relevant variant in exon 4 of POR gene: NM_001395413.1:c.253G>A; NP_001382342.1:p.Gly88Ser. The position was read with a depth of 41X, with the adenine at position 253 in 41 reads, compatible with a homozygous presentation. The variant was confirmed by Sanger sequencing in the patient and segregation analysis indicated that his mother was heterozygous for the same variant. Paternal DNA was not available (Fig. 2). The same homozygous variant was found in patients 2, 3, and 4 by Sanger sequencing, although a hemizygous presentation with a paternal allele deletion cannot be completely ruled out in cases 1 and 3. The sister of Patient 2 carried the same homozygous variant in the POR gene while both parents were heterozygous. Patient 3’s mother had the same variant in heterozygous state, and paternal DNA was not available. Both parents of Patient 4 were heterozygous for the same variant. The pedigrees show the inheritance pattern of the mutation, with the index cases (affected individuals) marked by arrows. The pedigrees also indicate the genotypes of the family members, with full black symbols representing individuals with the PORD phenotype and full white symbols representing those without the phenotype. The G88S and WT alleles are denoted by specific symbols.

The G88S variant was present in gnomAD exomes v4.1 (A = 0.0000143; 21/1613454) with rs782213151 (dbSNP v.157) and absent in gnomAD genomes and in our own in-house database including over 800 Argentine individuals. After considering in vitro functional studies performed here, showing variant characterization (see below), the variant was classified as *Pathogenic* (10 points) since it met the following ACMG/AMP criteria: PM1 (moderate, 2 points), PM2 (supporting, 1 point), PM3 (supporting, 1 point), PP3 (moderate, 2 points), PS3 (strong, 4 points).

### Glycine 88 Is Located Near FMN Binding Site in POR, and Mutation G88S Creates Protein Instability

**Figure 3.**
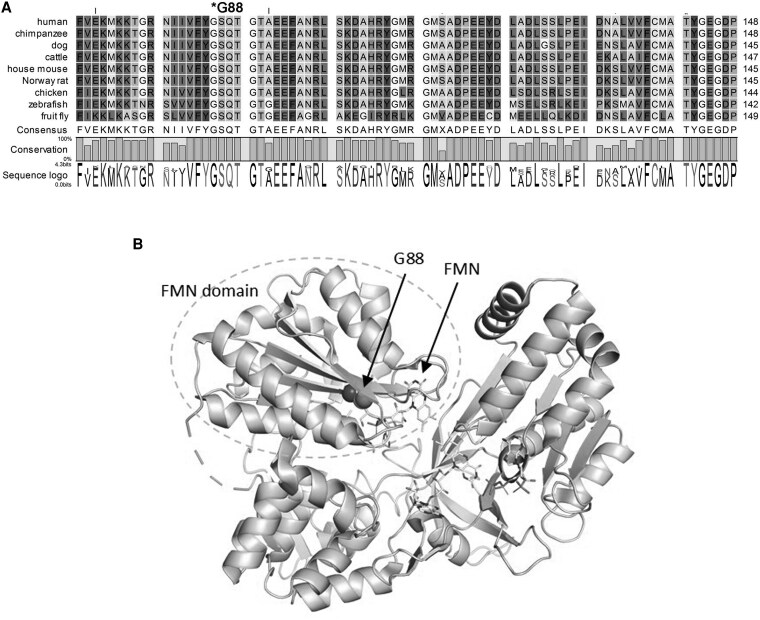
Sequence conservation and location of G88 residue in POR. A, Conservation of Glycine 88 (G88) in P450 oxidoreductase (POR) across different species. A multiple sequence alignment of a conserved region of the POR protein from various species, including human, chimpanzee, dog, cattle, house mouse, Norway rat, chicken, zebrafish, and fruit fly. The alignment shows a high degree of conservation of glycine 88 residue across evolutionarily diverse species. B, A structural model of the human POR protein. The location of the Glycine 88 residue, which is substituted by Serine in the G88S mutation, is indicated. The G88S mutation site is located in close proximity to the FMN binding domain of POR. This spatial relationship suggests that the G88S substitution may affect the binding and stability of the FMN cofactor, potentially disrupting the electron transfer function of POR.

Human POR has distinct domains for the binding of NADPH/FAD and FMN. The FMN binding domain interacts with the redox partners and is required for electron transfer to partner proteins (94, 95). The redox equivalents for the electron transfer are provided by NADPH which is used as a substrate by POR and converted to NADP. The G88S mutation causes the replacement of the amino acid glycine (G) with serine (S) at the 88th position of the POR protein. This substitution occurs within the critical FMN binding domain of the POR protein. The functional importance of the glycine residue at position 88 (G88) in human POR is underscored by its high degree of conservation across a wide range of species (Fig. 3A). This conservation, spanning from mammals to insects, suggests that this amino acid plays a critical role in the structure or function of the POR protein.

**Figure 4.**
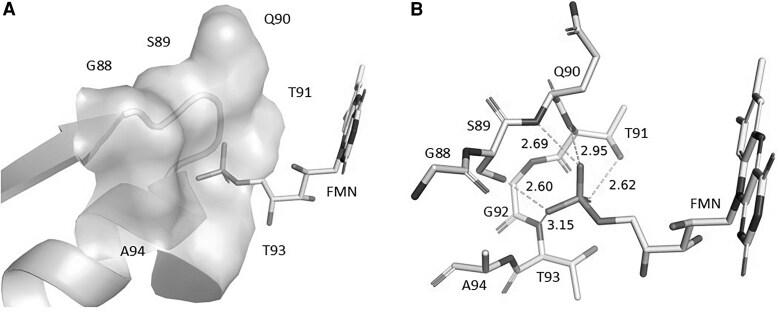
Interactions between POR-WT and the FMN cofactor. A, Structural representation highlighting the loop region (residues 88-94, depicted with surface) between β-strand 1 and α-helix B in wild-type human POR (PDB: 3QE2). This loop forms a cavity crucial for positioning the phosphate group of the FMN cofactor (shown in stick representation). Key residues within this loop (G88, S89, Q90, T91, G92, T93, A94) contribute to FMN binding. B, Detailed view of the hydrogen bonding network stabilizing the FMN phosphate group within the binding cavity. Specific hydrogen bonds (dashed lines) are shown between the phosphate oxygen atoms and the side-chain oxygen atoms of Serine 89 (S89) and Threonine 91 (T91), as well as the backbone nitrogen atoms of Glutamine 90 (Q90), Threonine 91 (T91), Glycine 92 (G92), and Threonine 93 (T93). Distances are indicated in Angstroms (Å). These interactions anchor the FMN cofactor in its functional position within the WT enzyme.

To obtain a more comprehensive understanding of how the G88S mutation may affect the protein's structure and function, we have analyzed the interactions between the protein and the cofactor FMN in the human wild-type POR (POR-WT) and in models of the mutant POR (POR-G88S). In the experimentally determined structure of human POR, the cofactor FMN is bound to the protein by both hydrophobic and polar interactions (Fig. 4). The isoalloxazine ring is sandwiched between Tyr143 and Tyr181 maximizing favorable π-π interactions, whereas the phosphate group is situated in a shallow cavity formed by residues in the loop between β-strand 1 and α-helix B (nomenclature according to Xia 2011 (91)). In this loop, the S89 and T91 side-chain oxygen atoms and the Q90, T91, and T93 backbone nitrogen atoms are all making hydrogen bonds to the terminal oxygen atoms in the phosphate group.

**Figure 5.**
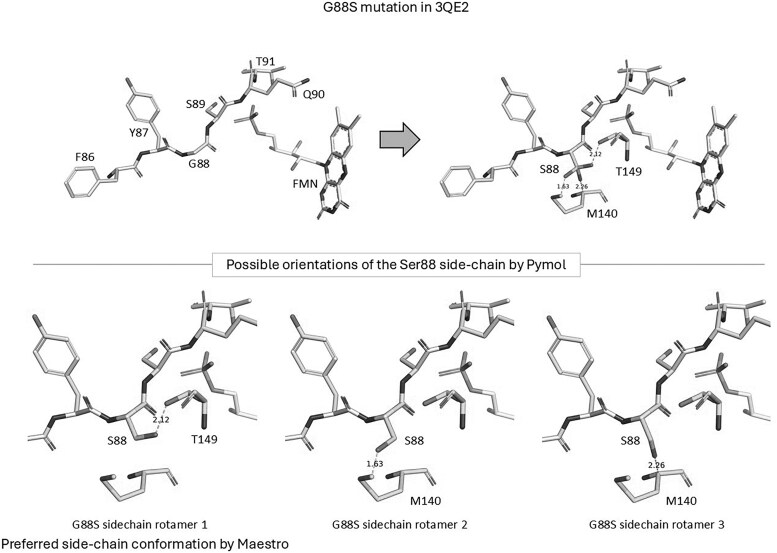
In silico mutagenesis modeling the G88S substitution in human POR. Structural comparison showing the local environment around residue 88 in the wild-type POR structure (PDB: 3QE2, left panel) and the modeled G88S mutant (right panel). The WT Glycine 88 (G88) is shown interacting with neighboring residues (F86, Y87, S89, Q90, T91) and the FMN cofactor. The right panel depicts the substitution with Serine (S88), illustrating potential steric or electronic changes introduced by the larger, polar side chain. Below, possible side-chain rotamers (conformations) for the introduced Serine 88 residue are shown as predicted by PyMol (top row) and Maestro (bottom row), highlighting the conformational flexibility and potential new interactions or clashes introduced by the mutation near the FMN binding site.

**Figure 6.**
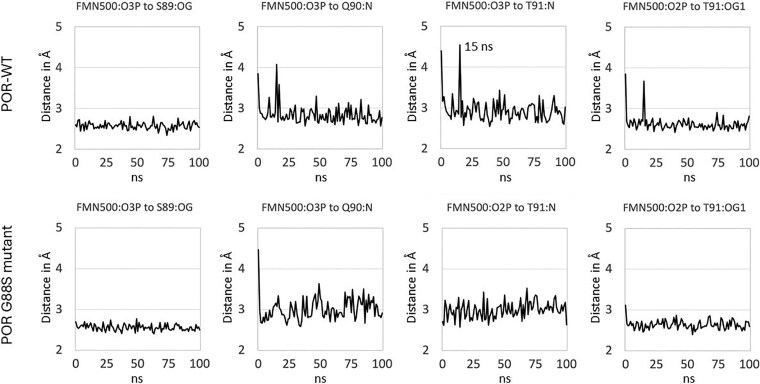
Stability of FMN interactions during molecular dynamics (MD) simulations. Plots showing key distances (in Å) between specific FMN phosphate oxygen atoms (O2P, O3P) and interacting protein residues (S89 side chain oxygen—OG, Q90 backbone nitrogen—N, T91 backbone nitrogen—N, T91 side chain oxygen—OG1) over the course of a 100 ns MD simulation. The upper row represents the simulation for POR-WT, showing relatively stable distances and hydrogen bonding patterns after initial equilibration. The lower row represents the simulation for the POR-G88S mutant, also showing relative stability in these specific interactions during the simulation, suggesting the closed conformation might retain FMN binding, although potentially weakened as suggested by dihedral angle changes (Fig. 7) and overall protein instability causing significant loss of flavins (Fig. 9). Note the transient increase in distance around 15 ns for the FMN:O3P to T91:N interaction in the WT simulation.

**Figure 7.**
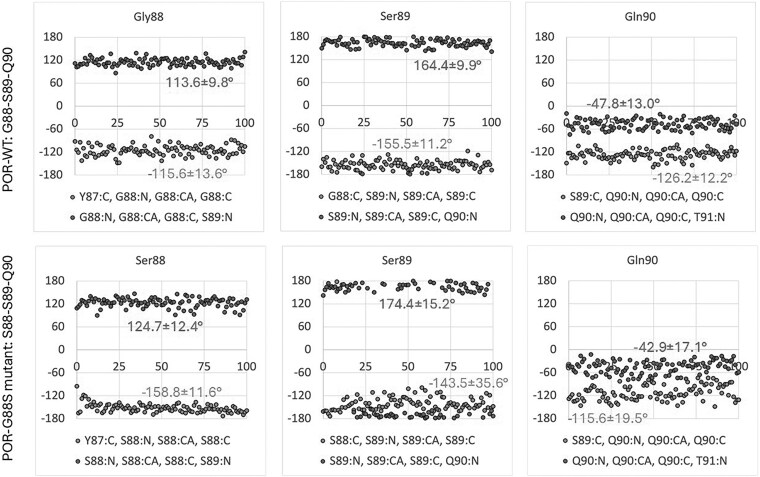
Backbone torsional angle variations in the FMN binding loop during MD simulations. Torsional angles of the ^88^GSQ^90^ loop in POR-WT (upper row) and the corresponding ^88^SSQ^90^ loop in the POR G88S mutant (lower row). Analysis comparing the phi (Φ, Cα-N bond rotation) and psi (Ψ, Cα-C bond rotation) backbone dihedral angles for residues 88, 89, and 90 in POR-WT (upper row) vs the POR-G88S mutant (lower row) over the 100 ns MD simulation is shown. Red dots represent Ψ angles, green dots represent Φ angles. Median values ± SD are indicated. The G88S mutation causes notable shifts, particularly an increase of >40° in the Ψ angle of residue 88 (comparing G88 Ψ ∼ −115.6° in WT vs S88 Ψ ∼ −158.8° in mutant) and smaller (∼10-20°) changes in other angles within the 88-SSQ-90 loop. These conformational alterations, although seemingly minor, may contribute to weakening FMN binding or altering loop dynamics, potentially affecting protein stability or function.

Comparison of the POR-WT structure and POR-G88S model reveals that there is no direct contact between the residue in position 88, either G88 or S88, respectively, and FMN. Mutation may often have an indirect effect, and we speculated on the effect of the glycine-88, being located at the end of a strand influenced the conformation of the subsequent loop comprising residues 88-94, which was altered due to G88S mutation (Fig. 5). We therefore performed MD simulations on the POR-WT and POR-G88S structures and looked for differences caused by the mutation (Fig. 6). MD simulation of POR-WT showed that binding of FMN and the hydrogen bonding pattern between the protein and FMN after an initial minor rearrangement remained stable during the simulation. The corresponding MD simulation of POR-G88S showed a nearly identical behavior with an initial minor rearrangement of the intramolecular hydrogen bonds followed by a stable system. Both MD simulations were characterized with smaller fluctuations for the hydrogen bonds involving the hydroxy sidechains of S89 and T91 compared to the fluctuations of the hydrogen bonds involving the backbone nitrogen atoms. To identify whether the mutation imposes an effect on the conformation of the loop, we compared the phi and psi angles of the residues 88-90 in POR-WT and POR-G88S (Fig. 7). The G88S mutation influences the backbone conformation of the loop with the position 88 psi dihedral angle increasing by more than 40° and the remaining dihedral changed by roughly 10°. Although this may not represent major changes, it may contribute to a weakening of the binding of FMN.

**Figure 8.**
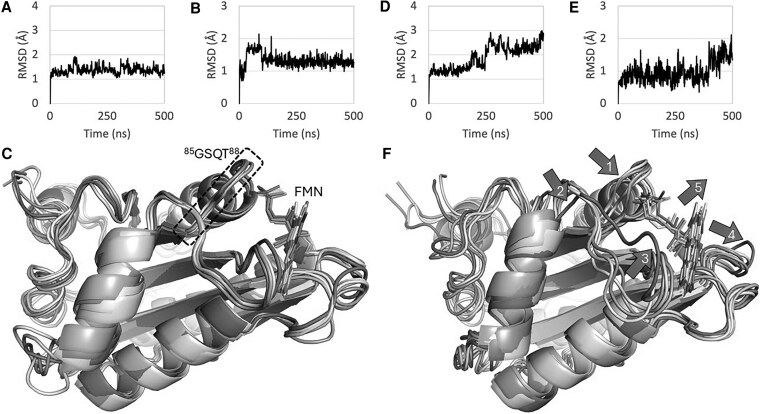
MD simulations of FMN domain in 3ES9 WT and 3ES9 G85S mutant (equivalent to human G88S). A-C, Data for rat 3ES9 WT. D-F) Data for rat 3ES9 G85S mutant. A and D, RMSD for protein Ca atoms. B and E, RMSD for ligand relative to protein. C and F, Overlap of structures after 100 (blue), 200 (cyan), 300 (beige), 400 (brown) and 500 (red) ns MD simulation. F, Arrows indicating the conformational changes relative to the initial structure: i) ^85^SSQT^88^ loop moved toward FMN (arrow 1); ii) ^140^GEGDPTDNA^149^ sequence underwent drastic conformational change (arrow 2 and 3); iii) ^209^DG^210^ loop moved away from FMN (arrow 4); and iv) FMN shifted slightly out of the originally defined binding pocket (arrow 5). Color figure is available in supplementary materials at https://doi.org/10.48620/91916 (65).

The effect of the G88S mutation may not necessarily be associated with a reduced flavin binding in the closed form of POR but could also be an upstream effect on the open form. The models for the open form of POR were based on the FMN domain extracted from the 3-dimensional structure of the open form of rat POR (PDB 3ES9) and the G85S mutant (corresponding to the G88S mutant in human POR). MD simulations of these 2 models showed that the FMN domain of the rat POR-WT was stable for the 500-ns simulation and that FMN, after a minor initial rearrangement, remained in the binding site as determined by x-ray crystallography (Fig. 8A-8C). The corresponding MD simulation of the rat POR G85S mutant showed that the protein made a conformational change after 250 ns, that the ligand subsequently changed its position relative to the protein after 400 ns and that several distinct conformational changes could be identified (Fig. 8D-8F).

**Figure 9.**
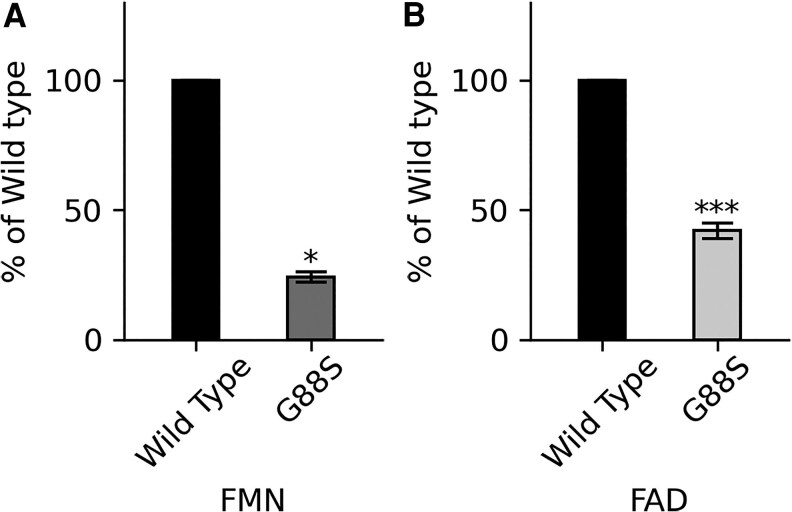
Flavin mononucleotide (FMN) and flavin adenine dinucleotide (FAD) content of wild-type (WT) and G88S mutant POR. Flavin content was determined by measuring the fluorescence of released FMN and FAD from purified WT and G88S POR proteins. The electron transfer from POR to its redox partners is mediated by its 2 obligatorily bound flavin cofactors, FAD and FMN. The G88S mutation in POR leads to a significant reduction in both FMN and FAD content, suggesting a potential mechanism for the impaired activity observed in individuals with this mutation. A, Bar graph showing the relative FMN content in G88S POR compared to WT POR. B, Bar graph showing the relative FAD content in G88S POR compared to WT POR. Statistical significance was determined by Student's *t* test to analyze the differences within experimental subsets, with *P < .05 and ***P < .001. Data are presented as mean ± SD from at least 3 independent experiments.

Based on the understanding of POR and the potential impact of mutations, the G88S mutation may affect POR in multiple ways: *Altered FMN binding:* The G88S mutation is located within the FMN binding domain (96). This mutation may disrupt the binding of FMN to POR, potentially affecting electron transfer. This disruption could occur due to changes in the shape of the binding site or alterations in the electrostatic interactions between FMN and POR. Although the G88 residue is located within the FMN binding region, the impact on both flavin cofactors (Fig. 9) suggests a broader consequence of the mutation on protein stability. *Reduced POR activity:* Impaired FMN binding or other structural changes caused by the mutation could reduce the overall activity of POR. This reduction in activity could result from a decrease in the efficiency of electron transfer or a disruption in the interaction between POR and its partner P450 enzymes. *Disrupted interactions with P450 enzymes:* The mutation may affect the interaction between POR and its partner P450 enzymes, leading to reduced P450 activity. This disruption could be due to changes in the binding interface between POR and P450 enzymes or alterations in the conformational dynamics of the POR-P450 complex.

POR mutations can be categorized based on their location and their impact on POR protein function. These mutations can be broadly classified into those that disrupt the FAD binding domain, those that affect the FMN binding domain, and those that impact the interaction surface between POR and P450 enzymes (1). Each of these mutation types can lead to distinct functional consequences for the POR protein and associated P450 enzyme activities. The FMN binding domain, where the G88S mutation is located, is crucial for POR's role as an electron carrier (97). POR utilizes FMN to transfer electrons from NADPH to P450 enzymes, enabling them to carry out their diverse catalytic functions. Disruptions in the FMN binding domain, such as those caused by the G88S mutation, can impair POR's electron transfer capabilities, leading to reduced P450 enzyme activity and potential downstream effects on steroidogenesis and drug metabolism.

To validate these predictions, we carried out a number of experiments to elucidate the specific functional impact of the G88S mutation on POR protein structure, electron transfer kinetics, and P450 enzyme interactions.

#### The G88S mutation in POR leads to a significant reduction in both FMN and FAD content

To investigate the impact of the G88S mutation on the flavin cofactor binding of POR, we measured the FMN and FAD content of purified recombinant WT and G88S POR proteins. The G88S variant exhibited a marked decrease in both flavin cofactors. The FMN content in the G88S mutant was reduced to approximately 30% of the WT level (Fig. 9A). Notably, the FAD content was even more severely affected, with the G88S mutant retaining only about 15% of the FAD levels observed in the WT protein (Fig. 9B). These reductions in flavin content were statistically significant (*P* < .05 for FMN and *P* < .001 for FAD).

While the direct impact of the mutation would likely be on the binding of FMN, the substantial reduction in FAD levels suggests a broader effect on protein stability and/or cofactor binding. One plausible explanation is that the G88S substitution alters the FMN binding domain, which may consequently affect the overall folding and stability of the protein. Such instability could lead to a decreased ability to retain both FMN and FAD, potentially through increased cofactor dissociation or protein degradation. The more pronounced reduction in FAD levels might indicate that the structural perturbation caused by the G88S mutation has a more destabilizing effect on the FAD binding environment, possibly through allosteric mechanisms or by affecting the interactions between the FMN and FAD binding domains within the POR molecule.

#### The G88S mutation in POR severely impairs its NADPH-dependent reductase activity toward various small molecule substrates

**Figure 10.**
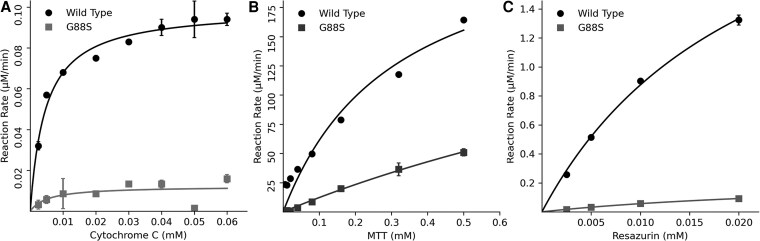
Kinetic analysis of small molecule reduction by wild-type (WT) and G88S POR. The NADPH-dependent reductase activity of purified WT and G88S POR was assessed using 3 different electron acceptor substrates. The reaction rates were measured at varying substrate concentrations, and the data were fitted to the Michaelis-Menten equation to determine the kinetic parameters. A, Cytochrome c reduction assay. The rate of cytochrome c reduction was monitored spectrophotometrically at 550 nm. The WT POR exhibited typical Michaelis-Menten kinetics, whereas the G88S variant showed a significantly reduced maximal velocity (Vmax) and potentially altered substrate affinity (Km). B, 3-(4,5-dimethylthiazol-2-yl)-2,5-diphenyltetrazolium bromide (MTT) reduction assay. The reduction of MTT to formazan was measured spectrophotometrically at 570 nm. Similar to cytochrome c reduction, the G88S POR displayed a markedly lower reductase activity compared to the WT enzyme across a range of MTT concentrations. C, Resazurin reduction assay. The reduction of the nonfluorescent resazurin to the fluorescent resorufin was monitored by measuring fluorescence (excitation at 560 nm, emission at 590 nm). The G88S POR showed a severely impaired ability to reduce resazurin compared to the WT POR, indicating a significant defect in its electron transfer capability to this substrate. These results demonstrate that the G88S mutation in POR leads to a substantial decrease in its reductase activity toward various electron acceptors.

The G88S mutation's impact on POR activity was evaluated by expressing both POR-WT and mutant POR-G88S in E. coli as N-23 forms and purifying bacterial membranes. The capacity of POR-WT and POR-G88S to transfer electrons from NADPH to cytochrome c, MTT, and resazurin was assessed. As depicted in Fig. 10A, the G88S POR variant exhibited a significantly decreased ability to reduce cytochrome c compared to the WT enzyme across a range of substrate concentrations. Kinetic analysis revealed a marked decrease in the catalytic efficiency (Vmax/Km) of the G88S mutant for cytochrome c, which was only 2.93% of the WT activity (Table 3). Similarly, the reduction of MTT (Fig. 10B) was also substantially impaired in the G88S variant, showing a catalytic efficiency of only 26% of the WT (Table 3). The most dramatic effect was observed in the resazurin reduction assay (Fig. 10C), where the G88S POR displayed a severely compromised activity, with a catalytic efficiency of only 8% of the WT (Table 3). These results collectively demonstrate a deleterious defect in the electron transfer capability of the G88S POR mutant.

**Table 3.** Kinetic parameters of wild-type and G88S POR for different substrates

	Vmax (nmol/min/mmol of POR)	Km (µM)	Vmax/Km (% of WT)
Cytochrome c reduction activity			
** WT**	78.1	4.6	16.9 (100)
** G88S**	19.9	39.5	0.5 (2.93)
MTT reduction activity			
** WT**	44.8	138.4	0.32 (100)
** G88S**	68	805	0.08 (26)
Resazurin reduction activity			
** WT**	2197	22	101 (100)
** G88S**	207	27	8 (8)

The table summarizes the kinetic parameters obtained for recombinant WT and G88S POR expressed in bacteria using cytochrome c, MTT, and resazurin as substrates.

Abbreviations: MTT, 3-(4,5-dimethylthiazol-2-yl)-2,5-diphenyltetrazolium bromide; POR, cytochrome P450 oxidoreductase; WT, wild-type.

The significant reduction in the reductase activity of the G88S POR mutant toward multiple small molecule substrates (cytochrome c, MTT, and resazurin) is consistent with the observed loss of flavin cofactors (Fig. 10) and likely contributes to the overall PORD associated with this mutation. As the G88S mutation is located in the FMN binding domain, the observed protein instability, as suggested by the reduced flavin content, could directly impact the efficiency of electron transfer from NADPH through FAD and FMN to downstream acceptors.

#### Steroid-metabolizing enzyme activity

**Figure 11.**
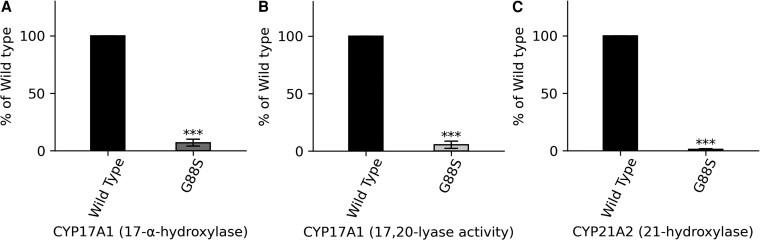
CYP17A1 and CYP21A2 activities supported by wild-type (WT) and G88S POR. The ability of WT and G88S POR to support the activities of 2 key steroidogenic cytochrome P450 enzymes, CYP17A1 and CYP21A2, was assessed in vitro. A, CYP17A1 17-α-hydroxylase activity, which catalyzes the conversion of pregnenolone to 17-hydroxypregnenolone and progesterone to 17-hydroxyprogesterone, is severely impaired when supported by G88S POR, showing only 7% of the WT activity. B, CYP17A1 17,20-lyase activity, which further converts 17-hydroxypregnenolone to dehydroepiandrosterone (DHEA), essential for the production of adrenal androgens, is also significantly reduced with G88S POR, exhibiting only 5.5% of the WT activity. (C) CYP21A2 21-hydroxylase activity, which is responsible for the conversion of 17-hydroxyprogesterone to 11-deoxycortisol (a precursor of cortisol) and progesterone to 11-deoxycorticosterone (a precursor of aldosterone), is most severely affected by the G88S mutation in POR, with the mutant supporting only 1.3% of the WT activity. Loss of CYP21A2 activity leads to CAH, characterized by impaired cortisol and aldosterone synthesis and an overproduction of androgens. Statistical significance was determined by Student's *t* test to analyze the differences within experimental subsets, with ***P < .001 compared to WT in all assays. Data are presented as mean ± SD from at least 3 independent experiments.

After observing decreased activity of the G88S POR mutant in reducing small molecule substrates, we investigated the impact of this mutation on the activity of key steroid-metabolizing cytochrome P450 enzymes, which rely on POR for their enzymatic function. To determine the functional consequences of the G88S mutation in POR on steroid hormone biosynthesis, we assessed the ability of the mutant protein to support the activities of CYP17A1 and CYP21A2, 2 crucial enzymes in the steroidogenic pathway. The G88S POR variant exhibited a dramatic reduction in its capacity to support the 17α-hydroxylase activity of CYP17A1, with the observed activity being only 7% of that supported by WT POR (Fig. 11A). Similarly, the 17,20-lyase activity of CYP17A1 was also severely affected, with the G88S POR supporting only 5.5% of the WT activity (Fig. 11B). The impact of the G88S mutation was even more pronounced on the 21-hydroxylase activity of CYP21A2, where the mutant POR supported a mere 1.3% of the activity observed with WT POR (Fig. 11C). These results indicate a major impairment in the ability of the G88S POR variant to transfer electrons to these key steroidogenic enzymes. These in vitro findings provide a direct molecular explanation for the patients' clinical biochemistry (Table 1). The devastating >98% loss of CYP21A2 activity correlates perfectly with the observed congenital adrenal hyperplasia (CAH)-like phenotype, while the >94% loss of CYP17A1 17,20-lyase activity explains the impaired androgen and estrogen synthesis central to the patients' gonadal dysfunction (Table 4).

**Table 4.** In vitro functional data for WT and G88S POR

Enzyme/activity	WT activity (% of WT)	G88S activity (% of WT)
*Flavin cofactor content*		
FMN content	100%	<30%
FAD content	100%	<15%
*Small molecule reduction*		
Cytochrome c reduction	100%	2.93% (catalytic efficiency)
MTT Reduction	100%	26% (catalytic efficiency)
Resazurin Reduction	100%	8% (catalytic efficiency)
*Steroidogenic CYP activities*		
CYP17A1 (17α-hydroxylase)	100%	7%
CYP17A1 (17,20-lyase)	100%	5.5%
CYP21A2 (21-hydroxylase)	100%	1.3%
*Drug-metabolizing CYP activities*		
CYP3A4	100%	3.3%
CYP3A5	100%	9%
CYP2C9	100%	3%
CYP2C19	100%	4%

A consolidated summary of the in vitro enzymatic activities of wild-type (WT) and G88S mutant P450 oxidoreductase (POR), presented as a percentage of the WT activity.

Abbreviations: FAD, flavin adenine dinucleotide; FMN, flavin mononucleotide; MTT, 3-(4,5-dimethylthiazol-2-yl)-2,5-diphenyltetrazolium bromide.

#### The G88S mutation in POR leads to a substantial reduction in the activities of major drug-metabolizing cytochrome P450 enzymes

**Figure 12.**
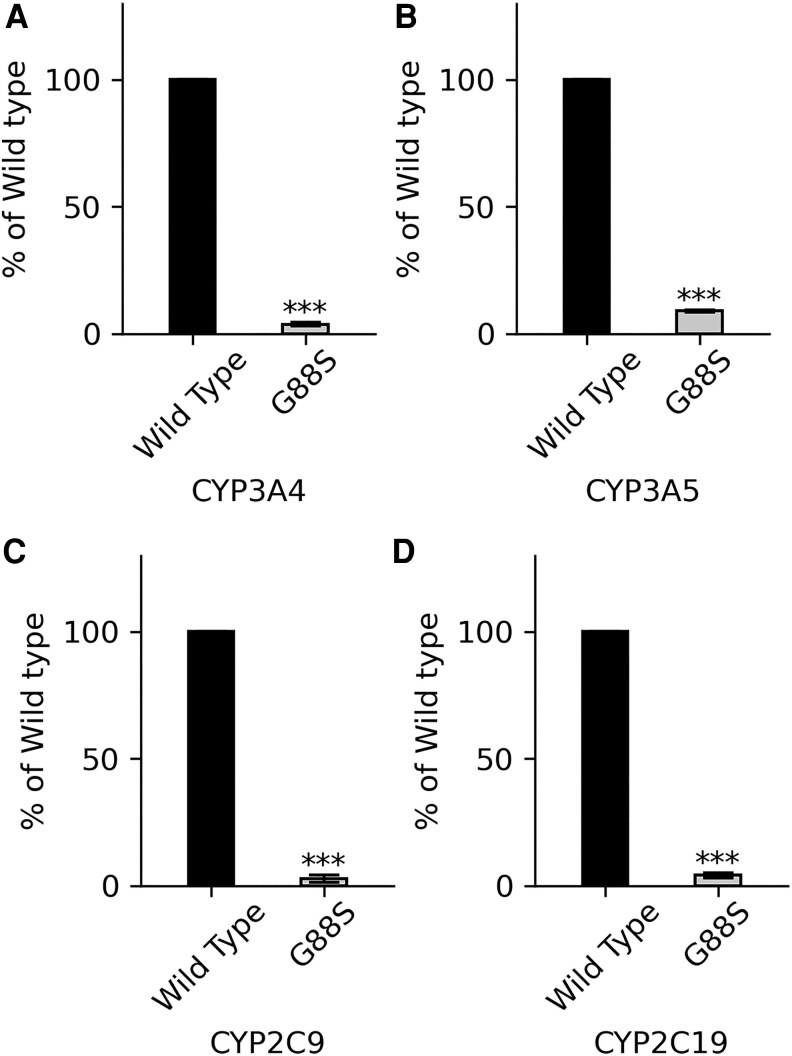
Activities of major drug-metabolizing cytochrome P450 enzymes supported by wild-type (WT) and G88S POR. A, CYP3A4, the most abundant drug-metabolizing enzyme in the liver, is responsible for the metabolism of approximately 50% of clinically used drugs, including statins (eg, atorvastatin, simvastatin), calcium channel blockers (eg, amlodipine, nifedipine), immunosuppressants (eg, cyclosporine, tacrolimus), and many others. The G88S POR variant supports only 3.3% of the WT CYP3A4 activity, indicating a severe impairment in the metabolism of numerous medications. B, CYP3A5 from the CYP3A subfamily with overlapping substrate specificity to CYP3A4, is more variable among individuals. The G88S POR variant supports 9% of the WT CYP3A5 activity, suggesting a significant reduction in its function. C, CYP2C9 is a major CYP2C subfamily enzyme involved in the metabolism of around 15% of clinically used drugs, including nonsteroidal anti-inflammatory drugs (NSAIDs) like ibuprofen and naproxen, the anticoagulant warfarin, and oral hypoglycemic agents like glipizide. The G88S POR variant supports only 3% of the WT CYP2C9 activity, indicating a substantial impact on the metabolism of these commonly prescribed medications. D, CYP2C19 is another key enzyme in the CYP2C subfamily involved in the metabolism of several important drugs, including proton pump inhibitors (PPIs) like omeprazole and lansoprazole, antiplatelet agents like clopidogrel, and some antidepressants and antiepileptics. The G88S POR variant supports only 4% of the WT CYP2C19 activity, highlighting a significant reduction in its capacity to metabolize these drugs. The severely compromised ability of the G88S POR variant to support the activities of these major drug-metabolizing enzymes underscores the potential for altered drug pharmacokinetics, including reduced drug clearance and increased risk of adverse drug reactions, in individuals carrying this mutation. Statistical significance was determined by Student's *t* test to analyze the differences within experimental subsets, with ***P < .001 compared to WT in all assays. Data are presented as mean ± SD from at least 3 independent experiments.

To investigate the impact of the G88S POR variant on drug metabolism, we assessed its ability to support the activities of several key human cytochrome P450 enzymes: CYP3A4, CYP3A5, CYP2C9, and CYP2C19. The CYP3A4 activity supported by the G88S variant was reduced to 3.3% of the WT level (Fig. 12A). CYP3A5 activity was also significantly impaired, with the G88S POR supporting only 9% of the WT activity (Fig. 12B). Similarly, the activities of both CYP2C9 and CYP2C19 were severely compromised, showing only 3% (Fig. 12C) and 4% (Fig. 12D) of the respective WT activities when supported by the G88S POR. These findings demonstrate a broad and significant impairment in the ability of the G88S POR mutant to facilitate the catalytic functions of major drug-metabolizing enzymes. The severe reduction in the activities of CYP3A4, CYP3A5, CYP2C9, and CYP2C19 observed with the G88S POR variant has significant implications for drug metabolism in individuals carrying this mutation. These 4 enzymes are responsible for the biotransformation of a vast array of clinically important drugs, collectively accounting for the metabolism of a large proportion of prescribed medications.

## Discussion

The G88S mutation in POR, identified in 4 unrelated Argentine families, is a missense mutation resulting in the substitution of glycine for serine at position 88. The clinical presentations of the 5 patients from 4 unrelated families homozygous for the G88S mutation reveal a crucial nuance in the understanding of PORD, demonstrating a textbook example of variable expressivity. While all affected individuals show the biochemical and genital abnormalities characteristic of severe PORD, a striking finding is the variable presence of skeletal malformations associated with Antley-Bixler syndrome (2-4, 8, 98). Patient 3 presents classic dysmorphic and skeletal features of Antley-Bixler syndrome, including arachnodactyly, thoracic kyphosis, and a bulbous nose. In contrast, the other 3 46,XY male patients, who share the identical G88S homozygous genotype, have no significant skeletal features of Antley-Bixler syndrome. This proves that the G88S mutation, while causing a severe biochemical defect, is not sufficient to produce the full Antley-Bixler syndrome skeletal phenotype on its own. This strongly suggests the existence of other modulating factors, such as genetic or environmental modifiers or subtle epigenetic influences, that contribute to the clinical expression of the disease. A significant finding of this study is the identification of the identical homozygous p.Gly88Ser mutation in 4 apparently unrelated families of Argentine origin. This variant is exceedingly rare in global population databases (gnomAD allele frequency ∼1.4 × 10^−5^), making its recurrence in a specific geographic cohort highly suggestive of a founder effect. While definitive proof would require haplotype analysis to demonstrate a shared chromosomal background among the affected individuals, the recurrence of this novel and severe mutation strongly suggests it may represent a regional founder allele. This has important clinical implications, suggesting that *POR* c.262G>A should be considered for targeted screening in Argentine patients presenting with DSD and/or biochemical evidence of combined CYP17A1 and CYP21A2 deficiency.

Furthermore, the case of the 46,XX patient (the sister of Patient 2) with delayed puberty, rather than the expected virilization, adds a crucial piece to the understanding of PORD's clinical spectrum. The in vitro assays demonstrated that the G88S variant severely impairs CYP17A1 17,20-lyase activity, reducing it to only 5.5% of wild-type. This enzyme is essential for gonadal androgen and estrogen production. The severity of this defect is so strong that it blocks both pathways, leading to the low estradiol levels observed in the patient and consequently, a failure of pubertal development. The elevated basal and stimulated levels of 17OHP and progesterone across all patients strongly correlate with the severely impaired CYP21A2 activity observed in vitro (Fig. 10C), where the G88S POR supported only 1.3% of WT activity. This leads to a block in the cortisol synthesis pathway, as reflected by the inadequately low or normal cortisol for the elevated ACTH levels. This finding reinforces the direct link between the molecular defect and the clinical presentation, highlighting the need for a nuanced understanding of PORD beyond the classic virilization phenotype (7, 30, 99-103).

The high degree of evolutionary conservation of this glycine residue, spanning from mammals to insects, underscores its critical functional importance. The substitution of this small, flexible amino acid with a bulkier, polar serine likely perturbs the delicate structural integrity of the FMN binding domain, leading to the molecular pathology observed. To elucidate the mechanism by which this localized mutation causes a widespread functional deficit, detailed structural and molecular dynamic analyses were performed. While in silico modeling of the closed POR conformation showed only minor changes in the in backbone angles, such as a greater than 40° shift in the psi (Ψ) dihedral angle of the 88th residue, with the FMN cofactor remaining in its binding pocket, a more powerful mechanistic explanation emerges from the dynamic simulations of the open conformation (104). Unlike mutations that may locally disrupt a static cofactor binding pocket, our simulations of the *open conformation* reveal that G88S fundamentally compromises the protein's essential dynamic flexibility. This dynamic failure propagates through the protein's structure, leading to global misfolding, an inability to properly bind *both* FMN and FAD, and subsequent degradation. This demonstrates that the mutation does not simply weaken cofactor binding in a static, stable protein, but rather fundamentally compromises the protein's dynamic flexibility, which is essential for its function. The substitution likely perturbs the protein's folding or maturation process, resulting in a large population of misfolded, flavin-deficient, and unstable protein that is then rapidly degraded. This is the definitive explanation for the severe loss of flavin cofactors observed experimentally (<30% FMN, <15% FAD).

To contextualize the severity of the G88S mutation, its functional profile was compared with that of other well-characterized POR mutations. A key precedent for protein instability is the L374H mutation, which affects a flexible hinge region of the POR protein and also causes a severe loss of flavin content (31). A quantitative comparison reveals that both mutations lead to a severe loss of enzymatic activity, but the G88S mutation appears to be at the extreme end of the severity spectrum, particularly for its devastating effect on CYP21A2 activity, which was reduced to a mere 1.3% of wild-type activity, compared to the 4% reported for L374H. Deficiency in CYP21A2 is the hallmark of classic CAH, characterized by impaired cortisol synthesis and a compensatory increase in ACTH secretion, leading to adrenal hyperplasia and excessive progesterone and 17OHP production. The severe impact of the G88S mutation on CYP21A2 activity likely accounts for the significant features of CAH often observed in patients with PORD (39, 42, 56, 105). The more severe impact of POR G88S on CYP21A2 (1.3% activity) vs CYP17A1 (5.5% activity) despite a global mechanism of protein instability, suggests that the residual, partially functional POR protein has a conformation that is particularly inept at supporting CYP21A2. It is established that different POR mutations can selectively impact partner enzymes; for instance, the common A287P variant preferentially inhibits CYP17A1 while retaining near-normal CYP21A2 activity. This highlights that the structural and dynamic requirements for each POR-P450 partnership are unique and highly specific. It can be hypothesized that the G88S-induced instability and conformational changes are exceptionally disruptive to the specific protein-protein interface or electron transfer dynamics required by CYP21A2, providing a direct molecular basis for the prominent CAH phenotype seen in all affected patients. The severe loss of steroid-metabolizing enzyme activities positions G88S as one of the most severe *POR* mutations described to date, establishing a new benchmark for the severity of a single homozygous mutation.

The in-depth functional characterization of the G88S POR variant extends beyond steroidogenesis to reveal serious implications for drug metabolism, a finding that has immediate and critical clinical relevance. The in vitro assays demonstrated that the G88S mutation severely impaired the activities of 4 major drug-metabolizing enzymes: CYP3A4, CYP3A5, CYP2C9, and CYP2C19. The activity supported by the G88S variant for each of these enzymes was reduced to a single-digit percentage of WT levels: 3.3% for CYP3A4, 9% for CYP3A5, 3% for CYP2C9, and 4% for CYP2C19.

A critical finding with immediate clinical relevance is that the G88S mutation creates a “poor metabolizer” phenotype, with residual activity for major drug-metabolizing enzymes like CYP3A4 and CYP2C9 falling to just 3% to 9% of wild-type levels. While no overt clinical evidence of adverse drug reactions was observed in this pediatric cohort, this is not unexpected given their young age and likely limited exposure to medications metabolized by these pathways. The absence of observed adverse drug reactions should not be interpreted as an absence of risk; rather, it underscores the profound importance of our in vitro findings as a preemptive pharmacogenomic warning. Studies in adult patients with other POR mutations have confirmed that such defects lead to impaired *in vivo* drug metabolism. For patients with the G88S mutation, standard doses of common drugs such as warfarin (CYP2C9), certain proton pump inhibitors (CYP2C19), or the vast number of prescription drugs metabolized by CYP3A4 could lead to severe toxicity. Therefore, the identification of this mutation is not merely a diagnostic tool for their endocrine condition but an essential, lifelong guide for personalized pharmacotherapy, mandating careful drug selection and dose adjustment to prevent future harm.

## Conclusion

The characterization of the G88S mutation in POR represents a significant advancement in the understanding of P450 oxidoreductase deficiency (Table 5). The in vitro functional assays, combined with molecular modeling, revealed a novel molecular pathomechanism: the G88S substitution causes a dynamic protein instability, leading to the catastrophic loss of both FMN and FAD cofactors. This widespread structural defect is the direct cause of the near-complete loss of enzymatic activity, particularly the devastating effect on CYP21A2, which was reduced to a mere 1.3% of wild-type function. This positions G88S as one of the most severe POR mutations described to date and provides a direct molecular link to the prominent CAH phenotype observed in all affected patients.

**Table 5.** Summary of molecular, functional, and clinical correlations of the *POR* G88S mutation

Molecular finding	In vitro functional consequence	Clinical/biochemical phenotype
Homozygous G88S mutation in FMN domain	Catastrophic loss of FMN and FAD (<30, < 15%) due to dynamic protein instability	Severe P450 oxidoreductase deficiency (PORD)
	CYP21A2 (21-hydroxylase) activity at 1.3% of WT	**Congenital adrenal hyperplasia:** High 17OHP & progesterone; Low/inadequate cortisol; High ACTH
	CYP17A1 (17,20-lyase) activity at 5.5% of WT	**Gonadal dysfunction:** 46,XY undervirilization (impaired testosterone); 46,XX delayed puberty (impaired Estradiol)
	CYP3A4/5, 2C9, 2C19 activity at 3%-9% of WT	**Lifelong pharmacogenomic risk:** “Pan-poor metabolizer” phenotype; High risk of adverse drug reactions (ADRs)

Abbreviations: 17OHP, 17-hydroxyprogesterone; ACTH, adrenocorticotropic hormone; FAD, flavin adenine dinucleotide; FMN, flavin mononucleotide; WT, wild-type.

The analysis of the clinical presentation further revealed a critical genotype-phenotype discrepancy. The identification of 4 individuals with an identical homozygous G88S mutation, but with only one of them presenting the full skeletal features of Antley-Bixler syndrome, indicates that the G88S mutation is necessary for the biochemical defect but not sufficient for the full skeletal phenotype. This strongly suggests that other genetic, epigenetic, or environmental factors are at play, modulating the clinical expression of the disease. Additionally, the case of the 46,XX patient with delayed puberty, rather than virilization, provides a new perspective on the clinical spectrum of PORD, demonstrating how the severity of a specific mutation can completely alter the expected phenotype by impairing key steroidogenic enzymes.

Beyond the established endocrinological effects, the findings on drug metabolism are of immediate and critical clinical utility. The G88S mutation effectively creates a “pan-poor metabolizer” phenotype for a vast range of commonly prescribed drugs by severely compromising the activities of CYP3A4, CYP3A5, CYP2C9, and CYP2C19. This information transforms POR from a gene of academic interest into a critical pharmacogenomic marker.

The characterization of the *POR* G88S mutation provides a clear example of translational medicine, linking a specific molecular defect to a severe clinical syndrome and a critical, lifelong pharmacogenomic risk profile. Based on these findings, genotyping for this specific variant should be considered in at-risk populations, such as Argentine newborns with DSD or suspected CAH. Early identification would not only enable timely and appropriate hormone replacement but would also allow for the entry of a preemptive pharmacogenomic warning into the patient's medical record. Future management of these individuals must involve close collaboration between endocrinologists and clinical pharmacologists. Further research should focus on developing a functional “activity score” for different POR variants against a panel of key P450 enzymes, creating a more nuanced predictive tool for both steroidogenic and drug-metabolizing capacity. Such an approach would pave the way for truly personalized medicine for all individuals living with PORD.

## Data Availability

All data are available in the manuscript or in the supplementary materials at https://doi.org/10.48620/91916 (65).

## Abbreviations

17OHP: 17-hydroxyprogesterone
ACTH: adrenocorticotropic hormone
ACMG: American College of Medical Genetics and Genomics
AMH: Anti-Müllerian hormone
CAH: congenital adrenal hyperplasia
DHEA-S: dehydroepiandrosterone sulfate
DSD: difference/disorder of sex development
EGS: external genitalia scoring
FAD: flavin adenine dinucleotide
FMN: flavin mononucleotide
FSH: follicle-stimulating hormone
hCG: human chorionic gonadotropin
LH: luteinizing hormone
MD: molecular dynamics
MTT: 3-(4,5-dimethylthiazol-2-yl)-2,5-diphenyltetrazolium bromide
NADPH: nicotinamide adenine dinucleotide phosphate
NGS: next-generation sequencing
POR: cytochrome P450 oxidoreductase
TLC: thin-layer chromatography
WT: wild-type
